# Investigation of Influences on Indoor and Outdoor SVOC Exposure

**DOI:** 10.3390/ijerph22040556

**Published:** 2025-04-03

**Authors:** Brianna N. Rivera, Lisa M. Bramer, Christine C. Ghetu, Diana Rohlman, Kaley Adams, Katrina M. Waters, Kim A. Anderson

**Affiliations:** 1 Department of Environmental and Molecular Toxicology, Oregon State University, Corvallis, OR 97331, USA; 2 Pacific Northwest National Laboratory, Biological Sciences Division, Richland, WA 99354, USA; 3 College of Health, Oregon State University, Corvallis, OR 97331, USA

**Keywords:** indoor air quality, semi-volatile organic compounds, passive sampling, chemical exposure, fragrance, community engaged research

## Abstract

Americans spend approximately 90% of their time indoors, with more than 66% of that time spent in residential buildings. Factors pertaining to household behavior or environmental factors may influence types of semi-volatile organic compounds (SVOC) found indoors. Paired indoor and outdoor passive samplers were deployed at twenty-four locations across the United States. Samples were analyzed for >1500 SVOCs to identify common patterns in exposure profiles and investigate influences of household behavior and environmental factors. Unique differences between indoor and outdoor profiles were identified, with indoor air typically having greater frequency and concentration of SVOCs relative to outdoor air. A significant relationship between fragrance chemicals and scented consumer products was identified. When considering a multifactorial approach, chemical exposures were most influenced by environmental and demographic factors. Our data highlights specific groups of chemicals identified at higher concentrations indoors and their potential influences, as well as the complexity of identifying specific sources of chemical exposures.

## 1. Introduction

There is a wide range of health effects associated with exposure to semi-volatile organic compounds (SVOC) relating to respiratory problems [1,2,3,4], adverse perinatal outcomes [2], reproductive and developmental disorders [5,6], neurotoxicity [7,8,9], immune system disruption [3,7], and increased cancer risk [7,10]. SVOCs are ubiquitously used in indoor environments via consumer products and building materials, yet are less widely studied [11,12,13]. These chemicals cover many chemical classes that are found in indoor and outdoor settings. The US EPA has more than 1000 SVOCs listed as high product volume chemicals [14]. These chemicals can be found in daily-use products such as plastic items, personal care products, cleaning products, furnishing, electronics, building materials, and everyday consumer pesticide formulations [11,12,15,16,17]. While SVOCs can be found in a number of commercial sources, they can also be produced as a by-product of indoor combustion such as burning candles, tobacco smoke, cooking, or grilling [18,19]. Due to their relatively high molecular weights and low vapor pressure, SVOCs tend to slowly release from their sources over a long period of time [12]. Additionally, due to their high surface/air partition coefficients, SVOCs tend to partition between the vapor phase and airborne particles that settle on dust and surfaces [11,20,21]. As a result of their slow release from sources and tendency to partition between phases, SVOCs can persist for years in indoor environments [12,22,23,24].

Geographic, sociodemographic, and behavioral variables are shown to be important influences on chemical exposures. For example, urban areas often have more sources of outdoor pollution compared to more rural settings [25]. Outdoor sources, such as proximity to roads, industrial facilities, hazardous waste, or Superfund sites, have been shown to have an adverse impact on indoor air quality [26,27,28,29]. Further, a correlation between race/ethnicity, socioeconomic status (e.g., % below the poverty line), or other factors—such as age of an individuals in a household or age of the home—were associated with increased proximity to Superfund sites, industrial emission sources, or number of industrial emissions sources [30,31,32].

Beyond geographic and demographic considerations, household behaviors, such as air ventilation and filtration, products used in the home, building materials, and cleaning habits, can influence concentrations or types of SVOCs found indoors. While increased ventilation has been reported to reduce indoor SVOC concentrations, some studies have shown that these may only have moderate impact or may result in intrusion of outdoor pollutants indoors [11]. The types of chemicals observed indoors may also be heavily influenced by the types of products in an individual’s home, such as furniture, paint, cleaning products, and personal care products [16,19,23,33]. Passive samplers are a useful tool to collect exposure information for SVOCs. They come in multiple configurations such as stationary or personal monitors, using polymers such as low-density polyethylene or silicone, respectively [34,35]. Stationary monitors are most often used to characterize chemicals present in the surrounding environment of specific environmental media (e.g., air, water, sediment) [34]. Both stationary and personal passive samplers are low-maintenance tools that non-selectively capture the bioavailable fraction of organic chemicals, which is representative of the fraction that would pass through the cellular membrane [36]. Concentrations of chemicals identified in these samplers represents a time-weighted average over a given deployment period. These samplers have previously been used to assess chemical exposure near unconventional natural gas drilling, oil spills, Superfund sites, firefighter exposures, and indoor and outdoor air quality during wildfires, among many others [37,38,39,40,41,42].

The objective of this exploratory study was to investigate indoor chemical profiles and the potential influences on these chemical profiles. To characterize the concentration and types of SVOCs in residences, we placed paired indoor and outdoor stationary passive sampling devices at twenty-four locations across the United States. A comprehensive chemical screening method was utilized to analyze samples for over 1500 SVOCs belonging to a wide range of chemical source categories [43]. Influences on exposure profiles were further investigated by incorporating publicly available sociodemographic and environmental factor data, or participant questionnaire data evaluating household behaviors.

## 2. Materials and Methods

### 2.1. Study Design

**Figure 1:**
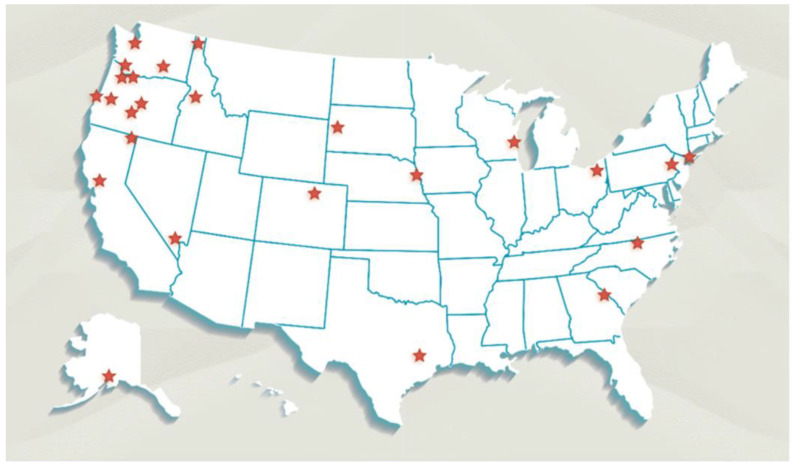
Each star represents a location where sampling took place. Paired indoor/outdoor sampling occurred at each location. Sampling took place at 24 locations, for a total of 48 combined indoor and outdoor samples.

This exploratory study used passive sampling devices, placed concurrently indoors and outdoors, at 24 residences across 15 states (Figure 1) for approximately 25 days (+/− 15 days) in the summer of 2019. Locations were a mix of urban, suburban, and rural locations (Table S1), based on the residential address provided by the participants.

Using a convenience sampling approach, participants (age 18 or older) were recruited from 1 April–30 June 2019 via existing community networks by telephone and email, and from within an existing cohort [38]. Initially, only environmental samples were collected. Shortly thereafter, we opted to collect sociodemographic and behavioral information via an optional survey (Table S2). The survey was reviewed and approved by the Oregon State University Institutional Review Board (protocol # IRB-2019-0312) and disseminated via Qualtrics (Qualtrics XM survey software, Provo, UT, USA). To ensure informed consent, participants were shown a multiple-choice question detailing possible survey activities. If participants selected the wrong responses, they were shown the study information again and allowed a second chance at answering the question. If they responded incorrectly twice, the survey was ended, and they were not able to continue with the survey portion of the study. Of the 24 participants, 19 (80%) opted to complete a survey, providing socio-demographic and behavioral information. One survey was returned without appropriate informed consent information, and the information was destroyed. The remaining 18 surveys were assessed to evaluate demographic and household behaviors (Table S3). All participants were given the option to receive a report back of their data, of which, all the participants opted to receive their results (Table S2). Reports back were provided to each participant with a summary of their results, including how their results compared to the rest of the study population, and background information on the different chemical classes evaluated.

Each participant was mailed a package containing nitrile gloves, a return label, two air cages with temperature loggers (HOBOware, Bourne, MA, USA), two sets of 5 LDPE strips in air-tight Teflon^®^ bags, deployment log sheets, and a detailed set of instructions on how to set up and take down the air samplers (Figure S1). Participants placed one stationary sampler inside and one outside their home, with requests to ensure the samplers were away from potential sources of SVOCs, such as the kitchen (emissions from cooking), outdoor grills or firepits, or vehicle exhaust. Multiple metrics were put in place to ensure participant compliance, including a check box denoting that gloves were worn prior to removing and when returning strips from the bag. Participants were instructed to leave samplers up for a total of 25 days, and a reminder email was sent prior to scheduled removal. Samplers were then taken down, placed in the provided Teflon ^®^ bag, sealed, and returned to Oregon State University (OSU). A total of 100% of samples were returned with boxes checked, indicating that all participants were wearing gloves when setting up and taking down sampling strips. Once returned to OSU, samplers were logged in and stored at −20 °C until ready to be cleaned and extracted, and average temperature for indoor and outdoor samplers was downloaded from each sampler using the temperature logger [38,39,44].

### 2.2. Sampler Preparation and Deployment

Prior to deployment, low-density polyethylene (LDPE) strips were conditioned to remove background contaminants using previously described procedures [40,42]. For each sampler, five LDPE strips were placed in air-tight Teflon ^®^ bags and stored at −20 °C until deployment.

### 2.3. LDPE Cleaning and Extraction

Samplers were cleaned to remove any residual dust or particles on the surface of the samplers by first rinsing with 18.2 MΩ-cm water, then isopropanol (Fischer Scientific, Waltham, MA, USA). Samplers were then cut into strips and stored in air-tight amber jars at −20 °C until extraction. Prior to liquid extraction, samplers were spiked with extraction surrogates at 500 ppm and extracted with n-hexane (Fisher Scientific, Waltham, MA, USA) as previously described [40,45,46]. Liquid extracts were then concentrated to 1 mL, aliquoted, and spiked with an internal standard, perylene-d12 (Cambridge Isotope lab, Andover, MA, USA). Samples were stored in amber glass vials at −20 °C until analysis.

### 2.4. Instrumental Analysis and Chemical Classifications

Sample aliquots were analyzed on an Agilent 7890 GC 5975C MS (GenTech Scientific, Arcade, NY, USA). This semi-quantitative method contains over 1500 individual analytes and uses a deconvolution software, Automated Mass Spectral Deconvolution and Identification System version 2.66 (AMDIS, NIST) [43]. Instrument parameters can be found in Table S4. While literature exists for the uptake rates of SVOCs, in this study we are constrained by the large variation in physical–chemical properties to accurately predict air concentrations. Therefore, reported concentrations of detected chemicals are expressed as nanomol/sampler. Protocols to ensure differences in time-weighted concentrations were accounted for, including instructing participants to deploy samplers for the same duration and only making comparisons between the same chemical.

Chemical categories in this method include polycyclic aromatic hydrocarbons (PAHs), pesticides, flame retardants, polychlorinated biphenyls (PCBs), industrial, pharmaceuticals, dioxins/furans, and personal care products (Figure S2). However, many of the chemicals within these categories may fall into multiple classifications. A list of all target chemicals in this method can be found at the following web address: http://fses.oregonstate.edu/1530 (accessed on 2 July 2022). Limits of quantitation for all target chemicals and more details about this method are further described in Bergman et al. [43]. Chemicals identified in this study, along with chemical structure classification, physicochemical properties, frequency of detection, and summary statistics of concentrations can be found in Table S5.

### 2.5. Quality Control

To ensure our samples met data quality objectives, quality control samples were included through this study during transport, processing, cleaning, and analysis on the instrument. Quality control samples include construction, trip, post-deployment, and instrument blanks. To demonstrate instrument precision and accuracy, sample duplicates and sample matrix spikes were prepared and analyzed. Quality control samples accounted for 23% of the total samples analyzed in this study. A continuing calibration verification (CCV) standard along with an instrument blank is run at the beginning and end of each batch. For a CCV to pass our data quality objectives (DQOs), greater than 60% of the target analytes must be identified within a factor of 2.5 times of the true value of the concentration of the CCV standard. All DQOs were met for the CCV, and extraction surrogate percent recovery (Table S6). Additional details on data quality objectives can be found in prior publications [41,43]. Diisobutyl phthalate was identified in one trip blank and two construction blanks. The average detected concentration in these blanks was used for background subtraction of all samples (Table S7).

### 2.6. Chemical Classifications

From the main chemical categories in the method (Figure S2), a narrower focus on chemical classifications was established for the chemicals identified in this study to better inform potential sources of these chemicals. Chemical source classifications were determined using the U.S. Environmental Protection Agency’s CompTox Dashboard (CTD) and the European Chemical Agency’s (ECHA) substance infocard [47,48]. One chemical, 1,2-dimethylnaphthalene, which had no information available on ECHA or CTD, utilized information from the good scents company for source categorizations [49]. Chemical source subcategories were identified based on source information for all chemicals. Source subcategories included building material, combustion by-product, consumer product, fragrance, furniture, personal care product, and pesticide (Table S8). Associated sources for each chemical category and a breakdown of which source subcategories fall into designated chemical categories in the method can be found in Table 1.

### 2.7. Environmental and Demographic Metadata

Regional locations (e.g., urban, suburban, rural) were defined using census tracts reported by the Center for Disease Control [50]. Locations were assigned as urban, suburban, or rural using population density as a measure of relative population distribution.

The United States Environmental Protection Agency’s tool EJ Screen was used to collect demographic data for each location [51]. Addresses of each sampling location, as provided by the participant, were used, and a five-mile radius was set for collection of environmental and demographic indicators. Environmental and demographic indicators and their associated sources can be found in Table S9. Square footage of homes was also collected via a search with each location’s address using Zillow.

### 2.8. Statistical Analysis

#### 2.8.1. Univariate Statistics

To evaluate the difference between indoor and outdoor chemicals measured, two statistical tests, based on mean concentration and detection probability, were conducted for each chemical. For each chemical with at least two indoor/outdoor sites with observed concentrations above the limit of detection (LOD), chemical concentrations were evaluated for a difference in mean concentration between indoor and outdoor locations. This resulted in a total 22 chemicals that were included in this comparison (Table S10). Measurements were log2 transformed, and values that were below the limit of detection (LOD) were assigned a value of NA to ensure the assumption of normality was not violated. A mixed-effects linear model with conditional normal distribution was fit for each chemical. Log_2_ chemical concentrations were used as the dependent variable. A fixed effect for indoor/outdoor location and random effect for sampling site were included in the model. A Benjamini–Hochberg multiple test adjustment was used. Chemicals with an adjusted *p*-value less than or equal to 0.05 were deemed to be statistically significant [52].

For each chemical with sufficient observations (three or more observed values) in either indoor or outdoor samples, a test for differences in detection rates between outdoor and indoor samples was conducted. This resulted in a total of 52 compounds included in this comparison (Table S10). A mixed-effects generalized linear model with conditional binomial distribution and random effect for sampling site was fit for each chemical. A likelihood ratio test was used to test for significant differences, and a Benjamini–Hochberg multiple test adjustment was used. Chemicals with an adjusted *p*-value of 0.05 or less were denoted as statistically significant.

Univariate analyses testing was conducted for significant associations between individual chemicals and questionnaire and environmental and demographic metadata. A linear model was fit with the response variable of chemical concentrations above LOD and each metadata variable as the explanatory variable individually. A generalized model with a conditional binomial distribution was fit with the binary detected/below LOD response variable and each metadata variable as the explanatory variable individually. For all tests and models, a Benjamini–Hochberg *p*-value adjustment across all chemicals tested was run to control the false discovery rate [52].

#### 2.8.2. Multivariate Statistics

Projection pursuit principal component analysis (PPCA) was run using log2 transformed chemical concentrations for each sampling site. This method provides the benefit that missing data does not need to be imputed for the algorithm to run [53].

Regression trees were fit with each chemical’s concentrations as the response variable and all metadata variables included as potential predictor variables [54]. Regression trees provide the advantage of being able to handle data with more predictors than observations, perform variable selection, and provide variable importance scores, and do not make any underlying distributional assumptions. Indoor and outdoor concentrations were modeled separately, and models were fit to chemicals with at least 25% of concentrations above the LOD. Observations below the LOD were substituted by ½*LOD, as regression trees do not assume data to follow a parametric distribution. The R package *rpart* was used to fit the models with the number of observations to consider a split set to 4 and using leave-one-out cross-validation to determine the optimal depth to which a tree should be grown [55]. Performance of each model was measured using the root mean squared error:

$$RMSE=\sqrt{\frac{\Sigma_{i=1}^{\;N}{\left(y_{i}-{\hat{y}}_{i}\right)}^{2}}{N}},$$

where N is the number of observed values, $y_{i}$ is the observed log_2_ concentration, and ${\hat{y}}_{i}$ is the fitted log_2_ concentration according to the model and R^2^ between observed and predicted concentration values. A null model was fit assuming the predicted value of all samples to be the mean of all observations, and the RMSE of this model (RMSE_null_) was recorded. Models with at least a 50% decrease in RMSE of the regression tree model compared to the null model and an R^2^ of at least 0.9 were denoted as high performing models for further evaluation (Tables S10 and S11). Variable importance metrics for each explanatory variable in the high performing regression tree were also evaluated.

## 3. Results and Discussion

### 3.1. Chemical Detection and Concentration

Of the over 1500 SVOCs each sample was analyzed for, 81 chemicals were detected in at least one sample across the indoor and outdoor samplers. These chemicals primarily belonged to chemical categories related to personal care products, industrial, or polycyclic aromatic hydrocarbons (Figure S2). The least number of detections were identified for chemical categories relating to pesticides, PCBs, dioxins/furans, pharmaceuticals, or flame retardants.

**Figure 2:**
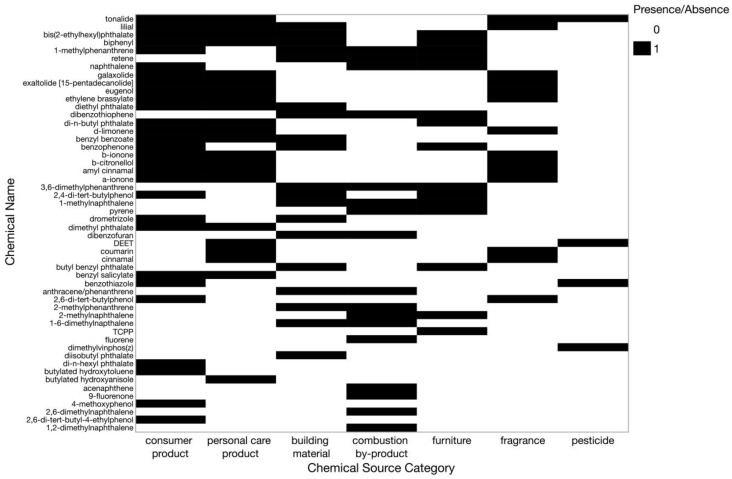
Chemical source categories for 52 of 81 total detected chemicals used for statistical comparisons. Only 52 chemicals had sufficient observations (three or more observed values) in either indoor or outdoor samples to be used for statistical comparisons. Black fill represents chemicals belonging to that chemical source category. Some chemicals were included in multiple source categories.

For statistical comparisons, only chemicals that had three or more observations indoor or outdoor were selected. This resulted in a total of 52 chemicals (Figure 2). Consumer products had the highest number of chemical detections, followed by personal care products and building materials. The pesticide source category had the least number of chemicals detected, with a total of four detections. Only one chemical, dimethylvinphos(z), was classified solely as a pesticide, whereas the others were also classified in the consumer product, personal care product, or fragrance source categories.

**Figure 3:**
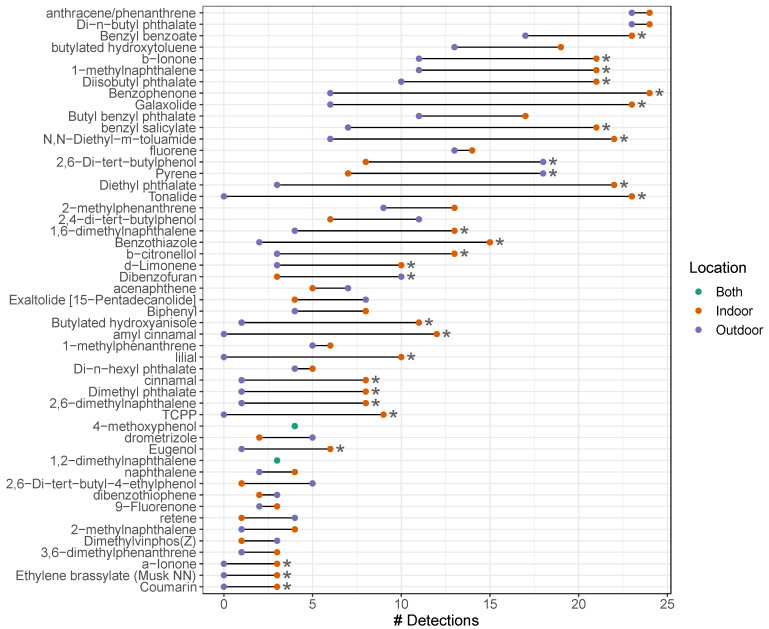
Number of times a chemical was detected above the limit of detection (LOD). Indoor (red point) vs. outdoor (blue point), sorted by total number of detections. In instances where the number of detections was the same for indoor and outdoor, a single green point was used (both). * Compounds with significant differences in number of observations indoor vs. outdoor.

Comparing indoor versus outdoor samplers, on average, more chemicals were detected indoors. On average, there were 23 detections per indoor sampler (range = 15–28) whereas outdoor samplers had an average of 13 detections (range = 6–31). As shown in Figure 3, there were 28 chemicals that were detected at a significantly greater frequency indoors compared to outdoors. Almost all the chemicals that had significant detections or higher concentrations indoors were related to consumer products or personal care products (Figure 2). Chemicals related to fragrance compounds and building material were unique to higher indoor detections and indoor concentrations, respectively. For example, benzyl benzoate was among the top three chemicals with significantly higher detections or concentrations indoors (Figure 3). β-ionone, 1-methylnaphthalene, n,n-diethyl-m-toluamide (DEET), and diethyl phthalate were also frequently detected, with significantly higher detections or concentrations indoors, respectively.

**Figure 4:**
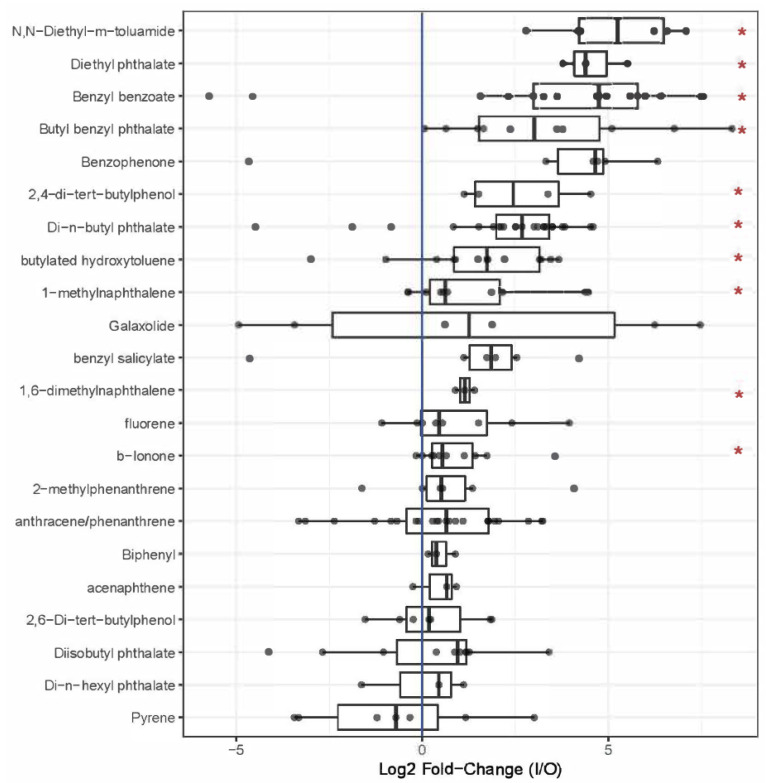
Paired log_2_ fold change for each sampling site (indoor/outdoor) by compound. Sorted by median concentration. * Compounds with significant differences in mean concentrations.

Similarly, chemical concentrations were higher indoors than outdoors. Specifically, ten chemicals had significantly higher mean concentrations indoors compared to outdoors (Figure 4). Overall, results showed significantly higher detections and concentrations of SVOCs indoors, with chemicals primarily belonging to consumer and personal care products source subcategories.

### 3.2. Influences on Chemical Profiles Univariate

#### Household Behaviors, Environmental and Demographic Influences

**Figure 5:**
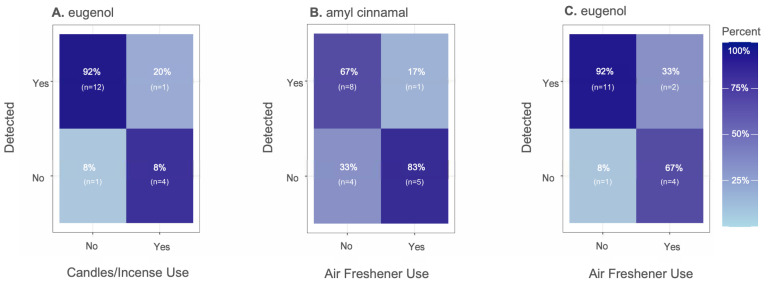
Number of fragrance chemical detections versus use of (**A**) candles/incense (**B**,**C**) air fresheners, expressed as a frequency plot. Darker colors indicate higher proportion of chemical detections, while lighter colors represent lower chemical detections. A significant relationship is seen if individuals without air freshener or candle/incense use have a higher percentage of no detection and individuals with air freshener/candle use have a higher percentage of chemical detection. Percentages are reported based on the total population that answered the survey.

Univariate analysis of associations between individual chemicals and questionnaire, environmental factors, or demographic metadata identified significant influences of household behavior, as well as demographic and environmental variables, on specific chemicals [56] The strongest relationship identified was between air freshener and candle/incense use with detections and concentrations of fragrance chemicals (Figure 5). These chemicals included eugenol, amyl cinnamal, b-citronellol, benzyl benzoate, galaxolide, and lilial. Detections of eugenol were higher for individuals who used air fresheners or candles/incense while amyl cinnamal had a moderate increase in detection frequency for individuals who used air fresheners (Figure 5). An increase in concentrations of benzyl benzoate, galaxolide, and lilial was also observed for residences that reported use of air fresheners compared to those who did not. Notably, the questionnaire data were able to explain increases in frequency or concentrations of fragrance chemicals in participants’ homes. Often there is limited confidence in the use of self-reported data due to issues relating to recall bias [57]. However, these findings validate the use of questionnaire data to predict chemical exposures with well-known sources such as fragrance chemicals.

### 3.3. Influences on Chemical Profiles Multivariate

#### 3.3.1. Household Behaviors, Environmental and Demographic Influences on Indoor Exposure Profiles

**Figure 6:**
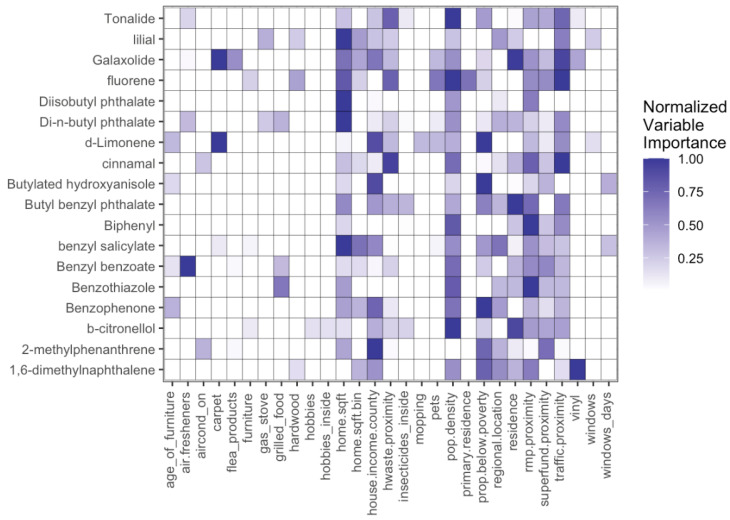
Normalized variable importance scores for each compound with high model performance and all variables selected by at least one model.

Regression trees were used in the multivariate analysis to identify relationships between chemical concentrations and questionnaire, demographic, or environmental metadata. Chemicals were identified as having a strong influence based on the variable importance factor (Tables S11 and S12). Population density, proximity to facilities with risk management plans (rmp), and traffic proximity had the strongest influence on indoor chemical concentrations (Figure 6).

Prioritization of chemicals using regression trees identified a total of 17 chemicals as being strongly influenced by these variables (Table S11). We focused on the top five chemicals with the highest variable importance factors, which included fluorene, cinnamal, tonalide, 1,6-dimethylnaphthalene, and biphenyl. After considering likely sources of these chemicals (defined in Table S8), fluorene and 1,6-dimethylnaphthalene are most likely to be related to environmental and sociodemographic variables. Reported emission sources of fluorene and 1,6-dimethylnaphthalene include coal tar, petroleum refineries or diesel exhaust, or tobacco smoke [58,59]. Through this process, we identified that indoor concentrations of fluorene and 1,6-dimethylnaphthalene were most likely to be influenced by proximity to rmp facilities, traffic, or population density. However, the remaining 15 chemicals identified cannot be ruled out. Thus, these findings suggest that chemicals related to outdoor sources are present in indoor spaces and may be impacting indoor air quality.

#### 3.3.2. Household Behaviors, Environmental and Demographic Influences on Outdoor Exposure Profiles

Population density, Superfund proximity, and traffic proximity had the strongest influence on concentrations of outdoor chemicals (Figure 6). A total of seven chemicals were identified as being most strongly influenced by these variables using regression trees (Table S12). The top five chemicals with the highest variable importance factors included diisobutyl phthalate, benzyl benzoate, b-ionone, 2,4-di-tert-butylphenol, and dibenzofuran. Chemical source subcategories of these chemicals include building material (diisobutyl phthalate, benzyl benzoate, dibenzofuran, 2,4-di-tert-butylphenol), consumer product (b-ionone, benzyl benzoate, 2,4-di-terat-butylphenol), personal care product (b-ionone, benzyl benzoate), fragrance (b-ionone), furniture (2,4-di-tert-butylphenol), or combustion by-product (dibenzofuran) (Figure 2). When considering potential sources of these chemicals, with the exception of dibenzofuran, it is unlikely that population density, Superfund, or traffic proximity are directly related to increased outdoor concentrations of these chemicals (Table S8). These chemicals are not combustion by-products, which would be expected for chemicals associated with proximity to traffic or Superfund sites. Rather, these chemicals are associated with building materials (diisobutyl phthalate, benzyl benzoate, 2,4-di-tert-butylphenol) and plant compounds (b-ionone).

**Figure 7:**
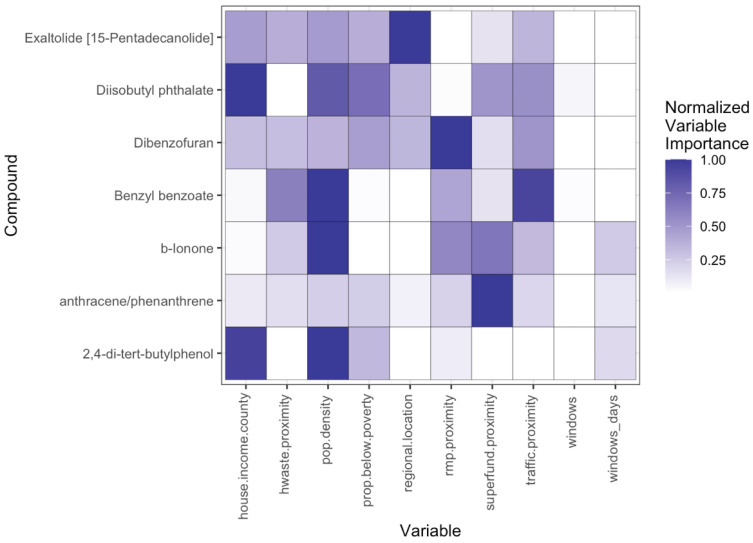
Normalized variable importance scores for each compound with high model performance and all variables selected by at least one model for outdoor measurements.

In contrast, dibenzofuran is a combustion by-product, with major sources of dibenzofuran to the general public being related to combustion of municipal waste or automobile exhaust (Figure 7) [60]. We predict that outdoor concentrations of dibenzofuran are most likely to be influenced by proximity to Superfund sites, traffic, or population density (as an indicator for increased proximity to traffic, industry, etc.). Thus, outdoor chemical detections are associated with potentially local features (buildings, vegetation) as well as known sources of pollution (traffic, Superfund sites).

#### 3.3.3. Household Behaviors, Environmental and Demographic Influences on Indoor/Outdoor Exposure Ratios

Ratios of chemicals with high model performance indoor and outdoor (*n* = 22) were investigated to explore the relationships between indoor and outdoor chemical concentrations and certain sociodemographic and environmental variables. Specifically, we assessed the potential for external sources (e.g., vehicle exhaust, industrial sources) to influence indoor air quality via influx.

Mean ratios of all identified chemical concentrations were higher indoor than outdoor (Table S13). Six of the twenty-two chemicals (fluorene, d-limonene, biphenyl, dibenzofuran, and exaltolide, anthracene/phenanthrene) had equal median concentrations indoor and outdoor or low mean concentrations (median ratio = 1 and mean ratio < 15), suggestive of influx. For the remaining chemicals, the higher indoor ratios suggest there are other factors, such as behaviors or household contributors that were not captured in our questionnaire, that may be influencing indoor concentrations of these chemicals.

Overall, we identified that a subset of six chemicals of the twenty-two that were identified as being influenced by sociodemographic or environmental factors, based on variable importance (Figure 6 and Figure 7; Tables S11 and S12), may be a result of an influx of outdoor chemicals indoors (Table S13). For the remaining chemicals, it is possible that there could be additional behavioral influences that were not captured in this study but could be correlated with these sociodemographic or environmental variables.

## 4. Conclusions

In this study, paired indoor/outdoor samples from across the United States were analyzed for over 1500 SVOCs from multiple chemical classes and sources. Comparisons between indoor and outdoor profiles identified unique differences between indoor and outdoor exposures. Indoor air had the highest number of detections for almost every location and predominantly higher concentrations. Of the chemicals identified in this study, chemicals with sources from consumer products, personal care products, and building materials were most frequently detected. Influences of household behavior and sociodemographic or environmental factors on exposure profiles were also investigated. Specific relationships between household characteristics and behavior such as use of scented consumer products (air freshener or candles/incense) in the household were identified, providing support for the utility of questionnaire data to predict chemical exposure for chemicals with well-known sources. When considering a multifactorial approach to identify the influences on chemical exposures, significant influences on chemical profiles were found to be most heavily influenced by environmental and demographic factors. These results highlight the complexity of identifying specific sources of chemical exposures. While results from this study were able to identify trends of sources and influence on chemical profiles indoors, the area of indoor air quality research would further benefit from a more comprehensive assessment to identify specific sources and ways to help reduce exposure.

## 5. Limitations

### Sampling Locations and Sample Size

We utilized passive samplers in a convenience sample to collect indoor and outdoor samples over 25 days across 15 states. Passive samplers are a low-cost, easy-to-use technology well-suited for characterizing semi-volatile and volatile organic compounds [35,44]. Here, we used an analytic method to investigate over 1500 chemicals [43]. Due to the diversity of physicochemical properties for the chemicals in this method, partitioning coefficients were not available for all chemicals, meaning that air concentrations could not be easily calculated. Therefore, concentrations were expressed as the concentration detected in the sampler (nmol/sampler), and samplers were deployed for similar periods of time to limit differences in concentrations as a result of partitioning over time.

We used a small convenience sample in this study. We had a higher rate of sampling in the Pacific Northwest (n = 12) relative to other regions, which is a limitation of our sampling approach. We also used self-administered questionnaires to collect information on household behaviors, demographics, and environmental influences. Approximately 75% of our questionnaire data were usable, meaning participants returned the questionnaire and appropriately answered the informed consent section of the survey. This resulted in a sample size of 18 out of 24 residences across the United States when looking at household behaviors and chemical profiles. We asked about household ventilation and the age of the home, but did not ask about the building type (e.g., single-residency home, apartment, etc.), nor did we ask for the specific room and square footage where the sampler was deployed. These questions should be included in future investigations as they may help inform understanding of indoor SVOC exposure. The results of this study are not anticipated to be representative of all exposures across the United States, but the trends we see are supported by other research on SVOC and VOC exposures [21,30,32,33].

## Figures and Tables

**Table 1:** Chemical source categories and associated sources for each category.

Chemical Source Category	Associated Sources
building material	paints, paints, coatings, adhesives, carpet, carpet padding, vinyl flooring
combustion by-product	vehicle exhaust, biomass burning, burning candles/incense, cooking or grilling
consumer product	children’s toys, shower curtains, cleaning products, detergents, air fresheners, lubricants, greases, clothing, polishes and waxes
fragrance	perfume, fragrance
furniture	fabric/vinyl upholstery
personal care product	sunscreen, shaving cream, hand soap, perfumes, cosmetics, toothpaste, deodorant, lipstick, shampoo, baby soap
pesticide	inert pesticide ingredients, pesticide

## Data Availability

Supplemental data in this study can be found in the Supplemental Materials. Supplemental data in the univariate analysis can be found on github. https://pnnl-superfund-research-center.github.io/Rivera_etal_Supplemental (accessed on 2 July 2022).

## Supplementary Materials

The following supporting information can be downloaded at https://www.mdpi.com/article/10.3390/ijerph22040556/s1; Table S1: List of sampling locations and associated regional distinction; Table S2: Survey results from participants that returned a paper or electronic survey; Table S3: Questions included in questionnaire, number of participants that answered each question, and percent compliance; Table S4: GC-MS Instrument Parameters RTL—retention time locking; Table S5. 81 Chemicals detected in indoor or outdoor samplers, chemical structural classification, physical-chemical properties, frequency of detection and mean, max and minimum concentrations detected; Table S6: Analyte used for background subtraction from samples; Table S7: List of extraction surrogates and average percent recovery; Table S8. Chemical Sources and Categories for 52 Chemicals Used for Statistical Analysis; Table S9. Participant demographic data collected from EJ Screen and Zillow [50,61] Table S10. List of chemicals used for qualitative and quantitative statistics; Table S11. Summary table of variable importance scores for indoor chemicals; Table S12. Summary table of variable importance scores for outdoor chemicals; Table S13. Summary statistics for Indoor/Outdoor Ratios for Chemicals with High performance metrics for the multivariate analysis; Figure S1. Instruction manual included in deployment package sent to participants, including pictures of sampling devices, sampling material, and bag used for sampler transport; Figure S2. Presence/absence of chemicals from each sample as defined by the original chemical categories first reported in Dixon et al., 2019 [62].
