# Supplementary material for: Investigation of Influences on Indoor and Outdoor SVOC Exposure
Source: Int J Environ Res Public Health. 2025 Apr 3;22(4):556. doi: 10.3390/ijerph22040556 (PMC12027231; doi:10.3390/ijerph22040556)
Supplement: Supplementary file 1 [file ijerph-22-00556-s001.zip › Supplemental.pdf]

SUPPLEMENTAL INFORMATION: INVESTIGATION OF INFLUENCES ON INDOOR AND OUTDOOR SVOC EXPOSURE

Brianna N. Rivera [1], Lisa Bramer[2], Christine C. Ghetu[1], Diana Rohlman[3], Kaley Adams[1], Katrina Waters[1,2], Kim A. Anderson [1]

[1]Department of Environmental and Molecular Toxicology, Oregon State University, Corvallis, OR 97331, USA;

[2]Pacific Northwest National Laboratory, Biological Sciences Division, Richland, WA 99354, USA;

[3]College of Health, Oregon State University, Corvallis OR 97331, USA

Table	Page #
**Table S1:** List of sampling locations and associated regional distinction	4
**Table S2:** Survey results from participants that returned a paper or electronic survey	5
**Table S3:** Questions included in questionnaire, number of participants that …	5
**Table S4:** GC-MS Instrument Parameters RTL – retention time locking	10
**Table S5**. 81 Chemicals detected in indoor or outdoor samplers…	11
**Table S6**: Analyte used for background subtraction from samples	15
**Table S7**: List of extraction surrogates and average percent recovery	15
**Table S8:** Chemical Sources and Categories for 52 Chemicals Used for Statistical Analysis	16
**Table S9.** Participant demographic data collected from EJ Screen and Zillow	16
**Table S10.** List of chemicals used for qualitative and quantitative statistics	17
**Table S11:** Summary table of variable importance scores for indoor chemicals	18
**Tabld S12**: Summary table of variable importance scores for outdoor chemicals	18
**Table S13.** Summary statistics for Indoor/Outdoor Ratios for Chemicals with…	18

Figures	Page #
**Figure S1.** Instruction manual included in deployment package sent to…	3
**Figure S2.** Presence/absence of chemicals from each sample as defined…	4

Instructions

Thank you for participating in the Indoor/Outdoor Air Quality study.

We are looking at differences in chemical exposure between indoor and outdoor air. A T-shaped metal box will be placed in your home and outside on your property for **three weeks**. These boxes contain passive sampling material that sample thousands of chemicals.

Thank you,
The Food Safety and Environmental Stewardship Lab

Diana Rohlman, PhD
College of Public Health and Human Sciences
P 541-357-8577 (*text ok*) E diana.rohlman@oregonstate.edu

Passive sampler set-up instructions

1. SET UP SAMPLER

Write the DATE & TIME you set up the sampler

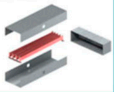

2. SAMPLE FOR 21 DAYS

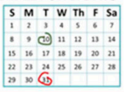

3. MAIL IT BACK

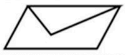

Write the DATE & TIME you remove the sampler. Put everything back in the box and use the provided label to mail it back!

Watch this video for a visual demonstration: https://youtu.be/M_j3HEbxIAY

1. Open the sampling box and it on a flat surface, free of dirt and debris.
2. Using gloves (provided) open the appropriate bag (Indoor / Outdoor)
   i. Use the wrong bag? Use a Sharpie to cross out and write in the appropriate location.
3. Remove a single strip. Place one loop over a post at the top of the box and wrap it around the opposite post on the bottom. Secure the strip to the second top post using a clip and a spring.

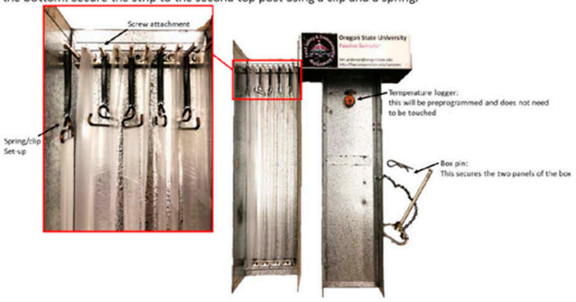

4. Repeat *four* more times on each of the screws until there are five strips in the box.
5. Label the bag, reseal it and place it back in the mailing box. Close the sampling box and replace the pin. You may now remove and throw away your gloves.
6. Repeat these steps for the next sampler box.
7. To remove the passive sampling strips, repeat these steps in opposite order.

Instructions

How to seal the bag

As illustrated in the picture below, your LDPE strips will come in a special plastic bag with a plastic closure (parts A & B). This bag prevents the strips from absorbing chemicals while it is in transit between you and our laboratory. **This bag is very important for the integrity of your strips.**

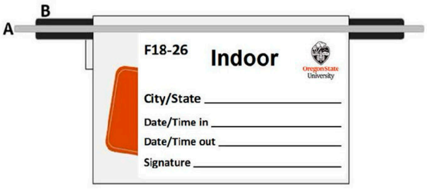

The bag may be resealed as follows:

1. Make one fold in the bag at the open end.
2. Place part **B** (the piece with a groove) on the backside of the fold and place part **A** (the thin plastic rod) on the front side and firmly press the two pieces together sealing the bag

Choosing a Location for the Air Box

**When placing the box, make sure that the bottom, open portion of the box remains open so that air can flow through.

Outdoors: Find an area of your property where the sampler box can stay without disturbing daily activities.

- Use zip ties (provided) and the openings on the back of the sampler to attach it to a tree, fence post or balcony.
- Alternatively, if you have a patio table/picnic table the box may lay flat on top.

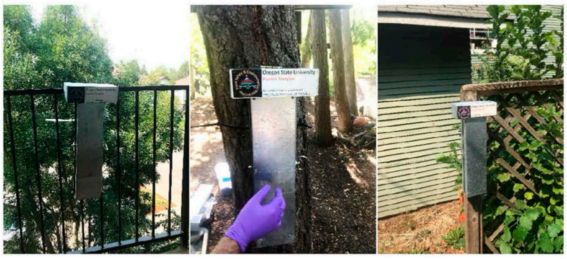

Indoors: Find an area of the room where the sampler can stay without disturbing daily activities.

- Use a drop cloth or old towel to protect furniture or the floor where the sampler box is placed.

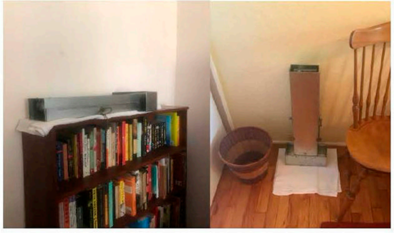

2 | Page

To learn more about our program please visit http://fses.oregonstate.edu

Figure S1. Instruction manual included in deployment package sent to participants, including pictures of sampling devices, sampling material, and bag used for sampler transport.

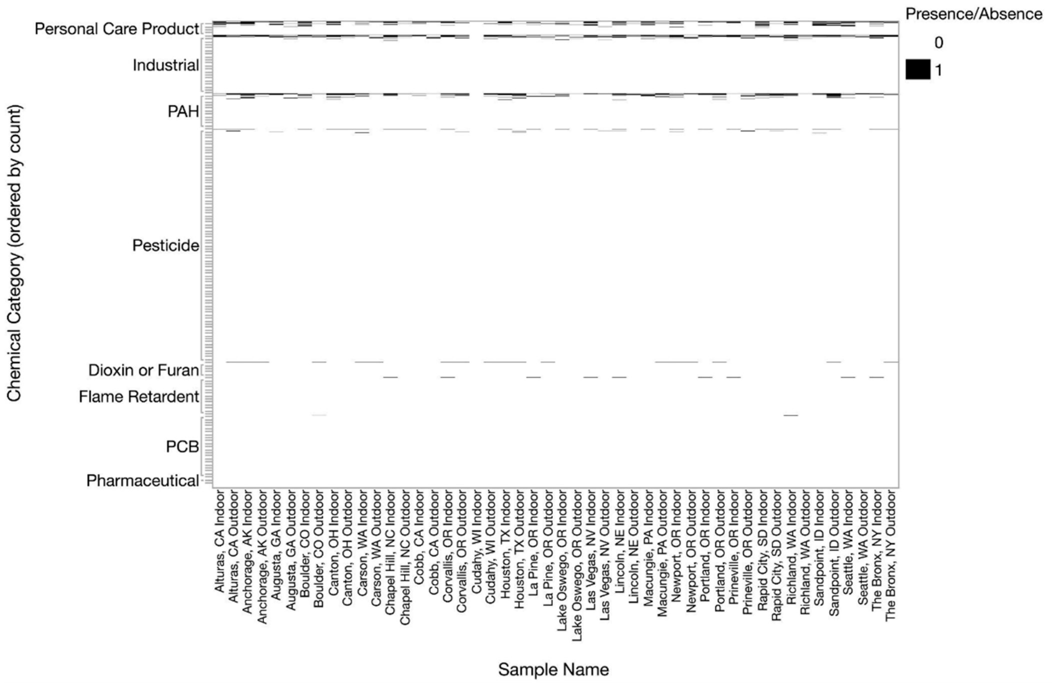

Figure S2. Presence/absence of chemicals from each sample as defined by the original chemical categories first reported in Dixon et al., 2019 [62]

Table S1: List of sampling locations and associated regional distinction.

Location	Region
Canton, OH	Midwest
Cudahy, WI	Midwest
Boulder, CO	Mountain West
Las Vegas, NV	Mountain West
Sandpoint, ID	Mountain West
Macungie, PA	Northeast
The Bronx, NY	Northeast
Anchorage, AK	Pacific West
Newport, OR	Pacific West
Corvallis, OR	Pacific West
Richland, WA	Pacific West
Alturas, CA	Pacific West
Seattle, WA	Pacific West
Portland, OR	Pacific West
Carson, WA	Pacific West
Lake Oswego, OR	Pacific West
Prineville, OR	Pacific West

Cobb, CA	Pacific West
La Pine, OR	Pacific West
Chapel Hill, NC	South
Augusta, GA	South
Houston, TX	South
Lincoln, NE	Midwest
Rapid City, SD	Midwest

Table S2: Survey results from participants that returned a paper or electronic survey.

SEE EXCEL FILE

Table S3: Questions included in questionnaire, number of participants that answered each question, and percent compliance.

**Question**	**%**	**N**
**1. Do you have an air conditioning unit?**		
YES	72%	13
NO	22%	4
No Response	6%	1
**1A. If YES, was it on during the study?**		
YES	69%	9
NO	31%	4
**1B. If YES, approximately how many days?**		
0 to 7 days	0%	0
7-14 days	22%	2
14-21 days	33%	3
21+	22%	2
No Response	22%	2
**2. Did you open your windows at any time during the study?**		
YES	44%	8
NO	50%	9

No Response	6%	1
**2A. If yes, approximately how many days?**		
0 to 7 days	38%	3
7-14 days	0%	0
14-21 days	38%	3
21+	25%	2
**3. Do you have hardwood floors?**		
YES	55%	10
NO	44%	8
No Response	0%	0
**4. Do you have carpet floors?**		
YES	67%	12
NO	33%	6
No Response	0%	0
**5. Do you have vinyl flooring?**		
YES	39%	7
NO	61%	11
No Response	0%	0
**6. Prior to or during the study did you have installations or remodeling done in your home/business? (i.e. paint, laminate flooring, cabinets, tile, new carpeting, etc.)**		
YES	11%	2
NO	78%	14
No Response	%	1
**6A. If yes, How recently did this remodeling occur?**		
0-3 months	50%	1
4-6 months	50%	1
7-9 months	0%	0
10+ months	0%	0
**6B. If yes, what kind of remodeling was done?**		

Carpet Replacement	50%	1
Paint	50%	1
**7. Do you have any hobbies which include the use of glues and adhesives, wood preservatives, or paints?**		
YES	17%	3
NO	72%	13
No Response	6%	1
**7A. If yes, do you conduct these hobbies inside your home/business?**		
YES	66%	2
NO	33%	1
**8. Approximately how many pieces of upholstered furniture (armchairs, couches) are currently in your home?**		
1-5	61%	11
6-10	33%	6
11-15	6%	1
**8A. Approximately how much of your furniture was purchased BEFORE 2004?**		
25%	67%	12
50%	16%	3
75%	6%	1
No Response	11%	2
**8B. Do you have plastic covers on your upholstered furniture?**		

YES	0%	0
NO	100%	18
**9. How many times a month do you mop?**		
1-5	89%	16
6-11	0%	0
11-15	0%	0
15+	0%	0
Not Applicable	11%	2
**10. Do you use air fresheners?**		
YES	33%	6
NO	67%	12
No Response	0%	0
**10A. If yes, how many do you have?**		
1-5	33%	2
6-11	0%	0
11-15	0%	0
15+	0%	0
**11. Were candles or incense burned during this study?**		
YES	28%	5
NO	72%	13
No Response	0%	0
**12. Do you have pets?**		
YES	78%	14
NO	22%	4
No Response	0%	0
**12B. Do you use anti-flea products?**		
YES	28%	4
NO	50%	7
No Response	22%	3

**13. Have chemicals been applied in your home/business to kill insects, either recently or since you have occupied your home/business?**		
YES	39%	7
NO	61%	11
No Response	0%	0
**14. Have pesticides ever been applied outside of your home/business?**		
YES	50%	9
NO	28%	5
No Response	22%	4
**15. Do you have a wood burning stove?**		
YES	11%	2
NO	78%	14
No Response	6%	1
**16. Do you have a gas stove?**		
YES	44%	8
NO	56%	10
No Response	0%	0
**17. Did you grill or charbroil food indoors during this study?**		
YES	11%	2
NO	83%	15
No Response	6%	1
**18. Do you or anyone in your home/business smoke?**		
YES	6%	1
NO	94%	17
No Response	0%	0

18A. If yes, do they smoke inside or outside your home/business?		
Inside	0%	0
Outside	6%	1

Table S4: GC-MS Instrument Parameters RTL – retention time locking.

GC-MS Settings		
Injection volume (µL)	1	
Mode	Pulsed Splitless	
Heater (°C)	265	
Pressure (psi)	RTL*	
Total flow (mL/min)	RTL*	
Septum purge flow (mL/min)	2	
System Carrier Gas	Helium	
Injection pulse pressure (psi)	25 until 0.5 min	
Purge flow to split vent (mL/min)	20 at 0.45 min	
Transfer line (°C)	300	
Column flow (mL/min)	RTL*	
Average velocity (cm/s)	RTL*	
**Temperature Program**		
**Time (min)**	**Ramp (°C/min)**	**Temp (°C)**
0	0	70
2	25	150
5.2	3	200
21.9	8	280
31.9	0	280
46.9	40	310
47.7	0	310
50.7	20	325
51.45	0	325
**Mass Spec Settings**		
Source temp(°C)	300	
Quad temp (°C)	150	
**Column Settings & Specifications**		
Column Type	Agilent DB-5MS	
Length (m)	30	
Diameter (µm)	250	
Film thickness (µm)	0.25	

Table S5. 81 Chemicals detected in indoor or outdoor samplers; chemical structural classification; physical–chemical properties; frequency of detection; and mean, max, and minimum concentrations detected.

CAS #	*Chemical Name*	*Chemical Structure Classification*	*Molecular Weight (g/mol)*	*Log Kow*	*Log Koa*	*Henry's Law*	*LOD (nmol/ sampler)*	*Frequency of Detection (%; n=48)*	*Mean (nmol/ sampler)*	*Min (nmol/ sampler)*	*Max (nmol/ sampler)*
573-98-8	1,2-dimethylnaphthalene	alkylated polycyclic aromatic hydrocarbon	156.2	4.31	5.892	6.41E-04	0.045	12.5	0.3	0.05	7
571-58-4	1,4-dimethylnaphthalene	alkylated polycyclic aromatic hydrocarbon	156.2	4.37	6.17E+00	0.000641	0.045	2.1	0.07	0.05	1.2
575-43-9	1,6-dimethylnaphthalene	alkylated polycyclic aromatic hydrocarbon	156.2	4.44	6.022	0.000641	0.045	35.4	0.69	0.05	5.1
90-15-3	1-hydroxynaphthalene	oxygenated polycyclic aromatic hydrocarbon	144.2	2.85	8.46	5.47E-08	0.098	2.1	0.83	0.1	35.4
90-12-0	1-methylnaphthalene	alkylated polycyclic aromatic hydrocarbon	142.2	3.87	5.55E+00	0.00058	0.05	66.7	1.85	0.05	19
832-69-9	1-methylphenanthrene	alkylated polycyclic aromatic hydrocarbon	192.3	5.08	7.78E+00	0.0000567	0.037	22.9	0.77	0.04	13
87-26-3	2-(1-methylbutyl)phenol	phenol	164.2	3.95	8.06E+00	1.92E-06	0.043	2.1	0.07	0.04	1.4
2772-45-4	2,4-bis(alpha,alpha-dimethylbenzyl)phenol	phenol	330.5	6.73	1.30E+01	1.38E-08	0.064	2.1	0.09	0.06	1.1
96-76-4	2,4-di-tert-butylphenol	phenol	206.3	5.19	9.01E+00	3.74E-06	0.069	35.4	6.02	0.07	111
59919-41-4	2,6-diethylnaphthalene	alkylated polycyclic aromatic hydrocarbon	184.3	5.25	6.59E+00	0.00113	0.038	2.1	0.06	0.04	0.9
581-42-0	2,6-dimethylnaphthalene	alkylated polycyclic aromatic hydrocarbon	156.2	4.31	5.89E+00	0.000641	0.045	18.8	0.53	0.05	7
4130-42-1	2,6-di-tert-butyl-4-ethylphenol	phenol	234.4	5.52	9.17	5.48E-06	0.06	12.5	0.1	0.06	0.7
128-39-2	2,6-di-tert-butylphenol	phenol	206.3	4.92	8.736	3.74E-06	0.069	54.2	1.39	0.07	5.3
939-27-5	2-ethylnaphthalene	alkylated polycyclic aromatic hydrocarbon	156.2	4.38	6.04E+00	0.000771	0.045	2.1	0.06	0.05	0.6

0613-12-7	2-methylanthracene	alkylated polycyclic aromatic hydrocarbon	192.3	5	7.64E+00	0.0000567	0.037	4.2	0.1	0.04	2.1
91-57-6	2-methylnaphthalene	alkylated polycyclic aromatic hydrocarbon	142.2	3.86	5.53E+00	0.00058	0.05	10.4	0.21	0.05	2.9
2531-84-2	2-methylphenanthrene	alkylated polycyclic aromatic hydrocarbon	192.3	4.86	7.50E+00	0.0000567	0.037	45.8	1.58	0.04	23.9
1576-67-6	3,6-dimethylphenanthrene	alkylated polycyclic aromatic hydrocarbon	206.3	5.44	8.03E+00	0.0000625	0.034	8.3	0.32	0.03	4.7
99-89-8	4-isopropylphenol	phenol	136.2	2.9	7.251	1.09E-06	0.104	2.1	0.16	0.1	2.9
150-76-5	4-methoxyphenol	phenol	124.1	1.58	7.45E+00	3.32E-08	0.228	16.7	0.62	0.23	3.8
84-65-1	9,10-anthraquinone	oxygenated polycyclic aromatic hydrocarbon	208.2	3.39	9.41E+00	2.35E-08	0.102	6.3	0.3	0.1	4
486-25-9	9-fluorenone	oxygenated polycyclic aromatic hydrocarbon	180.2	3.58	8.14E+00	0.000000677	0.078	10.4	1.55	0.08	37.2
83-32-9	acenaphthene	polycyclic aromatic hydrocarbon	154.2	3.92	6.04E+00	0.000282	0.046	25	0.49	0.05	6.5
127-41-3	a-ionone	ionone	192.3	3.85	5.98E+00	1.81E-04	0.11	6.3	0.46	0.11	11.4
122-40-7	amyl cinnamal	cinnamaldehyde	202.3	4.33	7.83E+00	7.80E-06	0.14	25	4.85	0.14	79.1
0120-12-7	anthracene	polycyclic aromatic hydrocarbon	178.2	4.45	7.09E+00	0.0000513	0.04	97.9	10.2	0.04	55
106-22-9	b-citronellol	monoterpenoid	156.3	3.91	6.544	5.68E-05	0.136	33.3	2.73	0.14	42.2
56-55-3	benz[a]anthracene	polycyclic aromatic hydrocarbon	228.3	5.76	9.069	0.00000501	0.031	2.1	0.04	0.03	0.3
119-61-9	benzophenone	benzophenone	182.2	3.18	7.281	1.94E-06	0.078	62.5	29.7	0.08	274
95-16-9	benzothiazole	benzothiazole	135.2	2.01	6.83E+00	3.74E-07	0.157	35.4	1.67	0.16	7.4
120-51-4	benzyl benzoate	benzoate ester	212.2	3.97	7.91E+00	2.80E-06	0.067	83.3	57.7	0.07	349
118-58-1	benzyl salicylate	salicylic acid benzyl ester	228.2	4.31	9.13E+00	3.67E-07	0.062	58.3	18.4	0.06	131

82657-04-03	bifenthrin	pyrethroid	422.9	8.15	12.538	1.91E-05	0.05	4.2	0.13	0.05	2.3
79-77-6	b-ionone	ionone	192.3	3.84	6.321	1.74E-04	0.074	66.7	8.28	0.07	57.2
92-52-4	biphenyl	biphenyl	154.2	4.01	5.91	4.14E-04	0.046	25	0.31	0.05	2
117-81-7	bis(2-ethylhexyl)phthalate	phthalate	390.6	8.39	1.17E+01	2.70E-07	0.054	100	32.2	5.12	179
85-68-7	butyl benzyl phthalate	phthalate	312.4	4.83	10.603	1.26E-06	0.091	58.3	17.8	0.09	256
25013-16-5	butylated hydroxyanisole	phenol	181.3	3.5	8.956	8.56E-08	0.117	25	1.79	0.12	13.2
128-37-0	butylated hydroxytoluene	phenol	220.4	5.1	8.87E+00	4.12E-06	0.064	66.7	10.7	0.06	63.5
99-49-0	carvone	terpenoid	150.2	2.71	5.21	7.73E-05	0.141	2.1	0.25	0.14	5.4
33704-61-9	cashmeran	alicyclic ketone	206.3	4.49	6.73E+00	1.42E-04	0.103	2.1	0.17	0.1	3.1
13171-00-1	celestolide	polycyclic musk	244.4	5.93	8.82E+00	3.18E-05	0.058	2.1	0.11	0.06	2.5
104-55-2	cinnamal	cinnamaldehyde	132.2	1.9	6.08E+00	1.60-006	0.16	18.8	3.43	0.16	59
5392-40-5	citral a	monoterpene aldehyde	152.2	3.45	5.26E+00	3.76E-04	0.093	2.1	0.16	0.09	3.4
91-64-5	coumarin	benzopyrone	146.1	1.39	6.78E+00	6.95E-06	0.194	6.3	3.28	0.19	130
52315-07-08	cypermethrin-2	pyrethroid	416.3	6.94	1.17E+01	7.89E-07	0.153	2.1	0.22	0.15	3.4
132-64-9	dibenzofuran	polycyclic aromatic hydrocarbon	168.2	4.05	6.825	4.11E-05	0.042	27.1	1.74	0.04	35.7
132-65-0	dibenzothiophene	polycyclic aromatic hydrocarbon	184.3	4.38	7.24E+00	0.0000279	0.038	10.4	0.7	0.04	21.2
84-66-2	diethyl phthalate	phthalate	222.2	2.65	7.44E+00	6.10E-07	0.064	52.1	71.3	0.06	1890
84-69-5	diisobutyl phthalate	phthalate	278.3	4.46	8.76E+00	6.43E-07	0.051	64.6	193	0.05	2147
11/3/0131	dimethyl phthalate	phthalate	194.2	1.6	6.69E+00	1.97E-07	0.073	18.8	7.06	0.07	283
67628-93-7	dimethylvinphos(z)	organophosphate	331.5	3.13	9.00E+00	2.93E-08	0.107	8.3	4.08	0.11	75.4
84-74-2	di-n-butyl phthalate	phthalate	278.3	4.5	8.63E+00	1.22E-06	0.051	97.9	79.9	0.05	255
84-75-3	di-n-hexyl phthalate	phthalate	334.4	6.57	1.04E+01	2.57E-05	0.042	18.8	0.45	0.04	4.2
84-76-4	di-n-nonyl phthalate	phthalate	418.6	9.52	12.59	1.41E-05	0.051	4.2	0.16	0.05	2.9
117-84-0	di-n-octyl phthalate	phthalate	390.6	8.54	1.19E+01	2.57E-06	0.036	2.1	0.1	0.04	3.1
5989-27-5	d-limonene	cyclic monoterpene	136.2	4.38	4.27E+00	3.80E-01	0.052	27.1	0.71	0.05	8.8
2440-22-4	drometrizole	benzotriazole	225.2	4.31	1.59E+01	6.12E-14	0.094	14.6	1.53	0.09	23.1

105-95-3	ethylene brassylate (musk nn)	macrocyclic lactone	270.4	4.71	8.60E+00	9.06E-08	0.262	6.3	2.28	0.26	88.8
97-53-0	eugenol	allylbenzene	164.2	2.27	7.98E+00	4.81E-08	0.173	14.6	1.91	0.17	26.2
0106-02-05	exaltolide [15-pentadecanolide]	macrolide	240.4	6.15	7.17E+00	2.32E-03	0.147	25	3.99	0.15	66.6
206-44-0	fluoranthene	polycyclic aromatic hydrocarbon	202.3	5.16	8.60E+00	0.0000083	0.035	2.1	0.04	0.03	0.4
86-73-7	fluorene	polycyclic aromatic hydrocarbon	166.2	4.18	6.59E+00	0.000167	0.043	56.3	1.81	0.04	24.1
1222-05-05	galaxolide	polycyclic musk	258.4	4.38	1.09E+01	6.89E-09	0.055	60.4	51	0.05	337
80-54-6	lilial	aromatic aldehyde	204.3	4.36	7.352	2.49E-05	0.138	20.8	10.8	0.14	108
78-70-6	linalool	monoterpene	154.3	2.97	6.03E+00	2.15E-05	0.137	2.1	0.62	0.14	23.3
93-15-2	methyleugenol	phenylpropene	178.2	3.03	6.67E+00	5.60E-06	0.158	2.1	0.18	0.16	1
134-62-3	n,n-diethyl-m-toluamide	monocarboxylic acid amide	191.3	2.18	8.25E+00	2.08E-08	0.111	58.3	31.5	0.11	476
91-20-3	naphthalene	polycyclic aromatic hydrocarbon	128.2	3.3	5.05E+00	0.000526	0.055	12.5	0.89	0.06	18.7
90-43-7	o-phenylphenol	phenol	170.2	3.09	7.457	1.05E-06	0.083	2.1	0.19	0.08	5.1
1825-21-4	pentachloroanisole	halogenated ether	280.4	5.45	9.58E+00	1.93E-03	0.126	4.2	0.38	0.13	11.1
52645-53-1	permethrin	pyrethroid	391.3	6.5	10.617	1.87E-06	0.054	4.2	0.12	0.05	2.8
85-41-6	phthalimide		147.1	1.15	7.53E+00	1.00E-08	0.24	2.1	0.94	0.24	34
51-03-6	piperonyl butoxide	benzodioxoles	338.4	4.75	1.32E+01	8.89E-11	0.063	2.1	7.46	0.06	355
129-00-0	pyrene	polycyclic aromatic hydrocarbon	202.3	4.88	8.19E+00	0.0000083	0.035	52.1	2.42	0.03	20.3
483-65-8	retene	alkylated polycyclic aromatic hydrocarbon	234.3	6.35	8.697	0.00011	0.03	10.4	0.69	0.03	15.4
13674-84-5	TCPP	organophosphate	327.57	3.11	8.723	5.96E-08	0.151	18.8	2.25	0.15	24.7
1918-11-2	terbucarb	phenyl methylcarbamates	277.4	5.28	10.296	2.36E-07	0.051	4.2	0.27	0.05	9.4
1506-02-01	tonalide	polycyclic musk	258.4	5.8	7.779	2.57E-04	0.055	47.9	6.22	0.05	81.3
115-86-6	TPP	organophosphate	326.3	4.7	8.46E+00	0.000000196	0.108	4.2	0.15	0.11	1.2
14324-55-1	zinc diethyldithiocarbamate	organosulfur	361.92	3.05	8.932	3.21E-08	390.323	4.2	374	0.09	390

Table S6: Analyte used for background subtraction from samples.

**Type of Sample**	**Diisobutyl Phthalate (nmol/sampler)**
Trip Blank	223
Construction Blank 1	126
Construction Blank 2	169
**Average**	172.6

Table S7: List of extraction surrogates and average percent recovery.

**Extraction Surrogate**	**Average Percent Surrogate Recovery (% RSD)**
Fluorenone-D8	254 % (31 %)
Acenaphthylene-D8	127% (5.0%)
Napthalene D-8	107% (28%)
Phenanthrene-D10	177% (27%)
Fluoranthene-D10	227% (28%)
Chrysene-D12	201% (29%)
Benzo(a)pyrene-D12	350% (180%)

Benzo(ghi)perylene-D12	83% (40%)
Tetrachloro-m-xylene (TCMX)	118% (27%)
PCB 100	146% (45%)
PCB 209	142% (40%)

Table S8: Chemical sources and categories for 52 chemicals used for statistical analysis.

SEE EXCEL FILE

Table S9. Participant demographic data collected from EJ Screen [51] and Zillow [52].

Sample Location	sq. ft of home	residence	primary residence	population density	Household Income by county	Residents who live below poverty line by county	Traffic Proximity (daily traffic count/distance to road)	Superfund Proximity (site count/km distance)	Hazardous Waste Proximity (facility count/km distance)
Alturas, CA	2510	1908		9112	45227	0.19	67	0.011	0.0088
Anchorage, AK	960	1953	1	301629	82113	0.1	700	0.19	1.4
Augusta, GA	2783	2003	1	136287	82184	0.07	370	0.14	1.2
Boulder, CO	1230	1968	1	310396	83591	0.1	890	0.12	3.8
Canton, OH	1512	1953		375222	52310	0.14	700	0.062	4.1
Carson, WA	1288	2003	1	22711	66607	0.11	100	0.022	0.036
Chapel Hill, NC	2430	2007	1	139365	71920	0.12	500	0.071	3.3
Cobb, CA	2927	2017		63843	45086	0.18	13	0.057	0.041
Corvallis, OR	2504	1972	1	85960	63600	0.16	180	0.099	0.68
Cudahy, WI	1902	1921	1	956386	49771	0.19	2000	0.074	4.3
Houston, TX	5595	1958	1	4352752	60241	0.17	1500	0.13	1.5
La Pine, OR	425	1978		165955	67372	0.09	26	0.0079	0.025
Lake Oswego, OR	1867	1975	1	388457	81061	0.07	710	0.12	1.9
Las Vegas, NV	3185	1999	1	2029316	57155	0.14	590	0.0047	2.3
Lincoln, NE	1130	1990	1	297285	58618	0.12	550	0.021	2.8
Macungie, PA	2807	2014	1	355768	62139	0.12	530	0.3	1.9
Newport, OR	2025	1965		46300	49300	0.14	480	0.016	0.016
Portland, OR	1200	1999	1	766082	70756	0.12	1400	0.12	4
Prineville, OR	1902	2006	0	20790	51348	0.13	0	0.0057	0.02
Rapid City, SD	944	1953		106372	58738	0.13	320	0.075	0.075
Richland, WA	3026	2010	1	184453	69212	0.1	390	0.076	0.15
Sandpoint, ID	1936	1980	1	40703	51373	0.14	260	0.014	0.54
Seattle, WA	642	2015	1	2046956	94822	0.09	2400	0.58	9.9
The Bronx, NY	650	2006	1	1427317	38566	0.27	3600	0.11	48

Table S10. List of chemicals used for qualitative and quantitative statistics.

Chemicals used for Qualitative Comparison	Chemicals used for Quantitative Comparison
1-methylnaphthalene	1-methylnaphthalene
1,6-dimethylnaphthalene	1,6-dimethylnaphthalene
2-methylphenanthrene	2-methylphenanthrene
2,4-di-tert-butylphenol	2,4-di-tert-butylphenol
2,6-di-tert-butylphenol	2,6-di-tert-butylphenol
acenaphthene	acenaphthene
anthracene/phenanthrene	anthracene/phenanthrene
β-Ionone	β-Ionone
benzophenone	benzophenone
benzyl benzoate	benzyl benzoate
benzyl salicylate	benzyl salicylate
biphenyl	biphenyl
butyl benzyl phthalate	butyl benzyl phthalate
butylated hydroxytoluene	butylated hydroxytoluene
di-n-butyl phthalate	di-n-butyl phthalate
di-n-hexyl phthalate	di-n-hexyl phthalate
diethyl phthalate	diethyl phthalate
diisobutyl phthalate	diisobutyl phthalate
fluorene	fluorene
galaxolide	galaxolide
N,N-Diethyl-m-toluamide	N,N-Diethyl-m-toluamide
pyrene	pyrene
tonalide	
benzothiazole	
b-citronellol	
amyl cinnamal	
butylated hydroxyanisole	
lilial	
dibenzofuran	
d-Limonene	
TCPP	
2,6-dimethylnaphthalene	
cinnamal	
dimethyl phthalate	
exaltolide [15-Pentadecanolide]	
eugenol	
1-methylphenanthrene	
2,6-Di-tert-butyl-4-ethylphenol	
drometrizole	
2-methylnaphthalene	
retene	
naphthalene	
4-methoxyphenol	
a-Ionone	
coumarin	

ethylene brassylate (Musk NN)	
3,6-dimethylphenanthrene	
dimethylvinphos(Z)	
9-Fluorenone	
dibenzothiophene	
1,2-dimethylnaphthalene	

Table S11. Summary table of variable importance scores for indoor chemicals.

SEE EXCEL FILE

Table S12. Summary table of variable importance scores for outdoor chemicals.

SEE EXCEL FILE

Table S13. Summary statistics for Indoor/Outdoor Ratios for Chemicals with High performance metrics for the multivariate analysis.

SEE EXCEL FILE
